# Seroprevalence and Risk Factors Associated with *Brucella* Infection in Camels in the Puntland State of Somalia

**DOI:** 10.3390/vetsci8070137

**Published:** 2021-07-19

**Authors:** Ahmed Said Mohamud, John Pilate Kothowa, Ruth Lindizyani Mfune, Melai Mubanga, Jacques Godfroid, John B. Muma

**Affiliations:** 1 Department of Disease Control, School of Veterinary Medicine, The University of Zambia, Lusaka P.O. Box 32379, Zambia; ruth.mfune@cbu.ac.zm (R.L.M.); jmuma@unza.zm (J.B.M.); 2 Faculty of Veterinary Medicine, Red Sea University, Galkayo RC2F+WC, Somalia; 3 Department of Animal Health and Livestock Development, BLADD, Mpemba P.O. Box 34, Malawi; kothowajohn@gmail.com; 4 Department of Public Health, Michael Chilufya Sata School of Medicine, The Copperbelt University, Ndola P.O. Box 71769, Zambia; 5 Department of Environmental Health, School of Medicine and Health Sciences, Eden University, Lusaka P.O. Box 37727, Zambia; melaimubanga@gmail.com; 6 Department of Arctic and Marine Biology, UiT—The Arctic University of Norway, 9037 Tromsø, Norway; jacques.godfroid@uit.no

**Keywords:** *Brucella*, camels, seroprevalence, Puntland State of Somalia

## Abstract

Brucellosis is an important zoonotic disease caused by members of the genus *Brucella*. Camel brucellosis has been reported in almost all camel-rearing countries in Africa and Asia. A cross-sectional study was conducted between February 2020 and February 2021 in Galkayo, Garowe, and Bosaso districts in the Puntland State of Somalia to investigate the seroprevalence and risk factors of brucellosis in camels. A competitive enzyme-linked immunosorbent assay (c-ELISA) was used to detect anti-*Brucella* antibodies, while a structured questionnaire was used to collect epidemiological data. A total of 441 camel sera were screened against *Brucella* antibodies. Thirty-one (7%; 95% CI: 4.8–9.8%) samples were positive, and thirteen (54.2%; 95% CI: 32.8–74.4%) out of the twenty-four farms sampled had at least one seropositive animal. Galkayo district had the highest number of *Brucella*-seropositive camels (10.3%), followed by Bosaso district (8.6%), while Garowe district had the lowest number of seropositive camels (1.4%). The binary logistic regression model revealed that camels in Galkayo district (*p* = 0.015; OR: 9.428; 95% CI: 1.539–57.755), camels from large herd sizes of >50 animals (*p* = 0.019; OR: 5.822; 95% CI: 1.336–25.371), and those in contact with small ruminants (*p* = 0.011; OR: 10.941; 95% CI: 1.728–69.285) were significantly associated with seropositive cases of camel brucellosis in the Puntland State of Somalia. The present study shows that *Brucella* infections in camels are prevalent in the three districts covered by the study. This poses a public health risk, because milk from these camels is used for human consumption. Studies focusing on the isolation of *Brucella* strains in camels and investigating brucellosis in ruminants and humans are recommended in the study area. Validation of serological tests—including c-ELISA—for *Brucella* antibody detection in camels is also needed.

## 1. Introduction

Somalia has the world’s largest dromedary camel (*Camelus dromedarius*) population, with more than seven million [1]. In addition to their social and cultural importance to Somali pastoralists, camels play a vital role in food security and national economy in the country. Interest in dromedary camel (one-humped camel) rearing has increasingly developed in arid countries, including Somalia, as dromedaries are more drought tolerant than any other domestic animals [2]. Camels are not only kept for milk, but also provide meat, and are used as a means of transportation. As a result of camels being the main source of milk in Somalia, intensification of camels for milk production in many parts of the country was started in the last few years.

Camels are, however, affected by a number of diseases, including brucellosis. Brucellosis is an important zoonotic disease caused by members of the genus *Brucella*. The disease can affect almost all domestic animals, and cross-transmission can occur between cattle, sheep, goats, and camels [2]. *Brucella* infection in camels is mainly caused by *B. abortus* and *B. melitensis*. Camels are not known to be the primary host for *Brucella* spp., and infection in camels depends on contact with other primary host animals [3]. Clinical disease in camels is very rare; however, infected camels are silent carriers of the *Brucella* pathogen, and the possible shedding of the organism in the milk may lead to transmission of the pathogen to humans [4,5,6]. Additionally, this disease imposes restrictions on the livestock trade.

Camel brucellosis has been reported in most camel-rearing countries in Africa and Asia [7]. In East Africa, camel brucellosis has been reported in Somalia, Ethiopia, Kenya, Sudan, and Eritrea [2]. However, there are no sufficient data about the real status of the disease in many countries of the world, and research on the epidemiology of camel brucellosis is very scarce [8,9]. Despite the fact that no test has been specifically validated in camels, a number of serological tests—including RBPT, ELISA, and the complement fixation test (CFT)—can be used for the diagnosis of camel brucellosis [10].

There is sufficient evidence indicating that brucellosis is present in sheep and goats [11,12], cattle [13,14], camels [3,15], and humans [16] in Somalia. However, there is currently little information available about the epidemiology of the disease in Somalia, and its occurrence in both livestock and humans is poorly estimated. Few studies have been conducted in south and central Somalia, or in Somaliland (northern Somalia) [3,15]. One study reported 3.9% individual seroprevalence in camels in northern Somalia [3], while 4.4% individual seroprevalence of camel brucellosis was reported in Mogadishu, southern Somalia [15]. However, no information is available on camel brucellosis in the Puntland State of Somalia (northeastern Somalia). According to the Food and Agriculture Organization of the United Nations (2014), livestock exports—especially sheep, goats, cattle, and camels—are the most traded commodity in Somalia. Brucellosis can hinder this huge livestock trade in the country.

Furthermore, many Somali people consume raw camel milk without heat treatment [15]; thus, *Brucella*-infected camels can pose health risks to the camel milk consumers in the country. Currently, there is no national control program for brucellosis in Somalia—a situation that might lead to spread of the disease among animals, resulting in huge economic losses to the livestock industry and negatively impacting public health. Therefore, this study was designed to investigate the seroprevalence and potential risk factors of brucellosis in camels in northeastern Somalia (the Puntland State of Somalia).

## 2. Materials and Methods

This study was conducted in the Puntland State of Somalia, a federal member state in northeastern Somalia. Puntland is bordered by Somaliland to its west, the Gulf of Aden to the north, the Indian Ocean to the east, the central Galmudug region to the south, and Ethiopia to the southwest. Puntland is semi-arid, with an average daily temperature range of 27–37 °C. The total area of the state is about 212,510 km^2^, with a population estimated at 4,334,633 in 2016 [17]. According to the Intergovernmental Authority on Development (IGAD) Centre for Pastoral Areas and Livestock Development (ICPALD), the estimated camel population stands at 1,868,000 in the Puntland State of Somalia as of 2013 [18]. Camels in Puntland State are mainly raised for milk and meat production under a nomadic system using traditional husbandry practices. However, commercialization of dairy camels, and keeping them in a semi-intensive production system, began in the last few years.

This cross-sectional study was conducted between February 2020 and February 2021, to determine the seroprevalence and risk factors of brucellosis in camels in the Puntland State of Somalia. Three districts were covered—namely, Bosaso (in the Bari region), Garowe (in the Nugal region) and Galkayo (in the Mudug region). Only commercially kept dairy camels were targeted, and these three districts were purposely selected because they represent the main dairy camel farming areas in the state. The number of animals to be sampled was calculated based on an assumed individual *Brucella* seroprevalence (unknown seroprevalence) of 50%, within a 95% confidence level and 5% desired precision. Sample size was obtained using the following formula [19]:

$$n=\frac{1.96^{2}\times \mathrm{Pex}\times \left(1-\mathrm{Pex}\right)}{{\left(d\right)}^{2}}$$

where n = sample size, d = desired absolute precision, and Pex= expected prevalence; thus, the desired sample size for Pex = 0.5 was n = 384. To account for non-response, a 10% adjustment was made, bringing the required sample size to 423 camels. However, at the end of the study, 441 camels were sampled.

A proportionally representative sample was allocated to each district based on its estimated number of dairy camel farms. A list of dairy camel farms in each district was obtained from the veterinary authority and from locals, and farms were selected randomly. All eligible animals were randomly sampled on every farm with ≤20 animals. For farms with larger herd sizes, a maximum of 30 animals were sampled from each herd. Each unvaccinated dairy camel commercially reared under the semi-intensive production system and above six months of age was included in the study. A structured questionnaire preloaded into smart phones using Epicollect5 (https://five.epicollect.net/, accessed on 8 December 2019) was administered in order to collect risk factors for brucellosis in camels. Information for each camel sampled was obtained, including age, sex, and history of abortion over the last two years. Information on each herd sampled was also obtained, including herd size, contact with ruminants, introduction of new camels to the herd, vaccination status, contact with other camel herds, and history of abortion in the herd over the last two years.

About 5 mL of whole blood sample was collected from the jugular vein, using plain vacutainer tubes and needles, from each camel. Sera were stored at −20 °C until being transported to the University of Zambia and tested via competitive enzyme-linked immunosorbent assay (c-ELISA). Sera samples were tested in singlicate. The c-ELISA test was conducted using the SVANOVIR^®^ Brucella-Ab c-ELISA kit and performed according to the manufacturer’s instructions. Any test sample with percentage inhibition (PI) < 30 was considered negative, and any test sample with PI > 30 was considered positive, according to the c-ELISA kit guide.

Data were analyzed using IBM SPSS Statistics 21. Seroprevalence and 95% confidence intervals (CIs) were computed. Potential risk factors associated with *Brucella*-seropositive cases at the individual animal level were initially screened via chi-squared test in univariate analysis. Moreover, all variables with *p* ≤ 0.25 in univariate analysis were used to construct a backward stepwise binary logistic regression model, and the degree of association was computed using odds ratios (ORs) and 95% confidence intervals (CIs).

## 3. Results

### 3.1. Distribution and Characteristics of Sampled Camels

A total of 441 camels in 24 herds from the Galkayo (185 camels), Garowe (140 camels), and Bosaso (116 camels) districts were examined. Of these, 346 (78.5%) were female, and 95 (21.5%) were male. The average herd size in the three districts was 65 animals per herd; 10 (41.7%) herds had <50 camels, while 14 (58.3) herds had >50 camels. The majority of the examined camels (251 camels) were older than 5 years (Table 1).

### 3.2. Seroprevalence of Camel Brucellosis

Out of the 441 tested serum samples, 31 (7%; 95% CI: 4.8–9.8%) were positive, while 13 (54.2%; 95% CI: 32.8–74.4%) of the 24 farms sampled had at least one seropositive camel. Regarding the seroprevalence of camel brucellosis in the three districts, Galkayo district had the highest number of c-ELISA-seropositive camels (10.3%), followed by Bosaso district (8.6%), while Garowe district had the lowest number of seropositive camels (1.4%) (Table 2).

### 3.3. Univariate Analysis of Risk Factors for BrucellaInfection in Camels

The results of the chi-squared cross-tabulation are presented in Table 3. Only variables with *p* ≤ 0.25 were further analyzed in the binary regression. No analysis was possible for contact with other camels, vaccination status, or ranging system, because all sampled camels were unvaccinated, had contact with other camel herds, and ranged freely.

In the multivariate logistic regression model at the individual level, Hosmer–Lemeshow goodness of fit test results showed that the model adequately fitted the data (X^2^= 0.229; *p* =0.994). The binary logistic regression model revealed that the camels in Galkayo district (*p* = 0.015; OR: 9.428; 95% CI: 1.539–57.755), camels from large herd sizes of >50 animals (*p* = 0.019; OR: 5.822; 95% CI: 1.336–25.371), and camels in contact with small ruminants (*p* = 0.011; OR: 10.941; 95% CI: 1.728–69.285) were positively associated with seropositivity for camel brucellosis in the Puntland State of Somalia (Table 4).

## 4. Discussion

The aim of this study was to describe the epidemiology of *Brucella* infection in semi-intensively kept dairy camels in the Puntland State of Somalia. We recorded a seroprevalence of 7%, and identified the area of production (districts), herd size, and contact with small ruminants to be major risk factors for *Brucella* infections in camels.

Based on the c-ELISA results, the individual seroprevalence of camel brucellosis in this study was estimated at 7%; this is the highest seroprevalence of camel brucellosis reported in Somalia. Despite the fact that there is no c-ELISA assay specifically validated in camels, the assay is widely used in the diagnosis of camel brucellosis [20]. Further, a study by Sayour et al. (2015) suggests that c-ELISA is an excellent test for the detection of *Brucella* antibodies in camels, but needs further standardization in camel sera [21]. The findings of the current study disagree with the studies conducted in southern northern Somalia [3,15], which reported lower seroprevalence of 3.9% using both SAT and c-ELISA, and 3.1% using i-ELISA, respectively. These results also contrast with studies reported in Ethiopia, where the seroprevalence was 2.2% [22], 4.1% [23], and 3.37% [24]. Seroprevalence of camel brucellosis higher than the seroprevalence found by this study (37.5%) was reported in Sudan [25]. It is known that the risk of the disease is higher in intensively reared animals due to increased exposure [26], and in this study, all sampled camels were from semi-intensively kept dairy camels, to which this relatively higher prevalence might be attributed. In addition to the lack of *Brucella* control programs in all domestic animals in Somalia, the free movement of camel herds and their contact with other herds and ruminants in the pasture and watering points can also contribute to the spread of *Brucella* infections. This finding shows that *Brucella* infection may pose a public health risk, as all sampled camels were apparently healthy animals from herds commercially reared for milk production.

The herd-level seroprevalence in this study was 54.2%. Few studies documenting herd-level seroprevalence of camel brucellosis have been conducted, and the majority of these studies reported high seroprevalence. For instance, 35.1%, 31.3%, and 10.23% herd-level seroprevalence were reported in Jordan, Somalia, and Ethiopia, respectively [9,15,27]. However, a herd-level seroprevalence of 1.5% was reported in Oman [4]. The present study focused on individual seroprevalence, and a small number of herds were sampled. Therefore, we recommend further studies on the herd-level seroprevalence of camel brucellosis in the study area.

The final binary logistic regression model in the present study revealed that, at the animal level, district, herd size, and contact with ruminants were significantly associated with camel brucellosis seropositivity.

District was statistically associated with *Brucella* seropositivity, whereby Galkayo district had a significantly higher seroprevalence of 10.3%, and camels in this district were about nine times more at risk of acquiring *Brucella* infection than camels in Bosaso district (*p* = 0.015; OR: 9.428; 95% CI: 1.539–57.755). Garowe district recorded relatively lower seroprevalence. These results corroborate those reported by Ghanem et al. (2009) in three districts in Somaliland, where the locality was significantly associated with the seropositive cases. Galkayo district is the capital of the Mudug region, central Somalia, which is bordered by Ethiopia to the west. Many camel herders who commercially kept camels in Galkayo district got their camels from Ethiopia, where the disease is endemic [28]. Moreover, the movement of camels between Somalia and Ethiopia is uncontrolled. These factors might explain this finding.

Farms with a large herd size (>50 camels) also showed statistically significant association with camel brucellosis, and were about six times more likely to have *Brucella* infection than farms with a small herd size (<50 camels) (*p* = 0.019; OR: 5.822; 95% CI: 1.336–25.371); similar observations have been reported by others [3,29,30,31]. In larger herds, more contact between camels may occur, especially during the calving season, which increases the chance of transmission of *Brucella* to susceptible camels.

Camels from herds that had contact with sheep and/or goats were also significantly associated with seropositivity for camel brucellosis, and were about 11 times more at risk than those who had contact with cattle (*p* = 0.011; OR: 10.941; 95% CI: 1.728–69.285). This suggests that *B. melitensis* may spill over from its small ruminant reservoir to camels, and might be due to the higher population density of sheep and goats in the study area, which share the pasture and watering points with camels, compared with the lower cattle population. The findings of our study are in agreement with those reported in Jordan [9].

## 5. Conclusions

The seroprevalence reported by the current study in the Puntland State of Somalia suggests that *Brucella* infection in camels is present in Galkayo, Bosaso, and Garowe districts. The risk factors influencing the seroprevalence of camel brucellosis at the animal level were district, herd size, and contact with ruminants. There is a need for further studies investigating brucellosis in ruminants and testing the correlation between brucellosis in camels and in ruminants. Studies focusing on the isolation of *Brucella* strains in camels in Somalia are also recommended. Moreover, serological assays, including c-ELISA, should be validated for *Brucella* antibody detection in camels. Further studies aiming at isolating *Brucella* spp. from camel milk would allow the tracing back of human infection in One Health approaches.

## Figures and Tables

**Table 1:** Distribution and characteristics of sampled camels.

No. of Sampled Camels				Herd Size		Age	
District	Female	Male	Total	Category	Frequency (%)	Age Group	Frequency (%)
Galkayo	166	19	185	<50 animals	10(41.7%)	<2 years	85(19.3%)
Garowe	100	40	140	>50 animals	14(58.3%)	2–5 years	105(23.8%)
Bosaso	80	36	116	-	-	>5 years	251(56.9%)
**Total**	346	95	441	**Total**	24(100%)	**Total**	441(100%)

**Table 2:** Seroprevalence of camel brucellosis.

Individual Level Brucellosis Seroprevalence			
**Sampled Camels**	**Positives**	%	**95% CI**
441	31	7%	4.8–9.8%
**Distribution of Brucellosis by Herd**			
**No. of Herds**	**Positives**	%	**95% CI**
24	13	54.2%	32.8–74.4%
**Distribution of Brucellosis by District**			
**District**	**Sampled Camels**	**Positives (%)**	**95% CI**
Galkayo	185	19(10.3%)	6.%3–15.6%
Garowe	140	2(1.4%)	0.2–0.5.1%
Bosaso	116	10(8.6%)	4.2–15.3%

**Table 3:** Risk factors for *Brucella* infection in camels at the individual level in univariate analysis.

Risk Factor	Category	*Brucella* Seropositivity		X^2^	*p*
		Negative	Positive		
District	Galkayo Garowe Bosaso	166 138 106	19 2 10	10.143	0.006
Introducing new camels	No Yes	214 196	7 24	10.111	0.001
Sex	Female Male	321 89	25 6	0.094	0.759
Age	<2 years 2–5 years >5 years	80 98 232	5 7 19	0.304	0.859
Herd size	<50 animals >50 animals	135 257	6 25	2.441	0.118
History of abortion in the herd	No Yes	268 142	20 11	0.009	0.924
Contact with ruminants	With sheep and goats With both small and large ruminants	123 287	14 17	3.049	0.079

**Table 4:** Binary logistic regression analysis of risk factors for camel brucellosis at the individual level.

Variable	ß ^1^	SE ^2^	*p*	OR ^3^	95% CI ^4^
**District**					
Bosaso	-	-	-	-	-
Galkayo	2.244	0.925	0.015	9.428	1.539–57.755
Garowe	0.345	1.202	0.774	1.412	0.134–14.903
Herd size (>50 animals)	1.762	0.751	0.019	5.822	1.336–25.371
Contact with small ruminants	2.393	0.942	0.011	10.941	1.728–69.285
Constant	−6.083	1.539	0.000	0.002	-

^1^ ß: regression coefficient; ^2^ SE: standard error; ^3^ OR: odds ratio; ^4^ CI: confidence interval.

## Data Availability

The data supporting the reported results can be made available on request from the corresponding author.
